# Ten-year hospitalization trends in Mexico: Examining the profile of national and transient and migrants

**DOI:** 10.3389/fpubh.2022.1060861

**Published:** 2023-01-25

**Authors:** René Leyva-Flores, Belkis Aracena-Genao, Nirma D. Bustamante, Ietza Bojorquez, Ricardo Cortés-Alcalá, Diana Gómez-López, Miguel Adonai Pérez-Sastré

**Affiliations:** ^1^Center for Research in Health Systems (CISS), National Institute of Public Health (INSP), Cuernavaca, Morelos, Mexico; ^2^Center for Research in Nutrition and Health (CINyS), National Institute of Public Health (INSP), Cuernavaca, Morelos, Mexico; ^3^Centers for Disease Control and Prevention, Atlanta, GA, United States; ^4^Migration and Health, El Colegio de la Frontera Norte, Tijuana, Baja California, Mexico; ^5^Ministry of Health (Secretaría de Salud), Mexico City, Mexico; ^6^Independent Public Health Researcher, Mexico City, Mexico; ^7^Faculty of Medicine, Universidad Nacional Autónoma de México (UNAM), Mexico City, Mexico

**Keywords:** hospitalizations, transients and migrants, health services accessibility, Mexico, Central America, South America, United States

## Abstract

**Aim:**

In Mexico, as in other societies, migrants are seen as over-users of health services. However, the extent, distribution, and trends of use over time are unknown. Evidence is needed to inform health policies and improve health services for foreign patients. The objective of this study was to examine factors associated with the distribution and trends of Mexican and foreign resident hospitalizations in Mexican public hospitals from 2010 to 2020.

**Methods:**

A graphical and statistical analysis (descriptive and correlational) of discharge trends in public hospitals was carried out. Hospitalization trends were analyzed by country of habitual residence (Mexico, US, Central and South America, and Other Continents), age, sex, primary discharge category, and region of service delivery. Adjusted Poisson modeling was used to examine the factors associated with annual hospitalizations of Mexican and foreign residents.

**Results:**

Between 2010 and 2020, there were 26,780,808 hospitalizations in Mexican public hospitals. Of these, 0.05% were of foreign residents. Hospitalizations for Mexican residents remained stable from 2010 to 2019, while those for foreign residents trended upward over the same period. In 2020, hospitalizations of Mexican residents fell by 36.6%, while foreign resident hospitalizations fell by 348.8%. The distribution of hospitalizations by sex was higher among females for all categories of habitual residence, except among US residents. Obstetric discharges were the most common reason for hospitalization among Mexican residents (42.45%), Central and South American residents (42.24%), and residents from Other Continents (13.73%). The average hospital stay was 2 days. Poisson regression confirmed these results, showing that hospitalizations was higher among women (except among foreign residents) and in the ≤ 17 age group. Poisson modeling also showed that trauma injury was the leading cause of discharge for foreign residents after obstetric causes.

**Discussion:**

It is unlikely the upward trend in hospitalizations among foreign residents in Mexico from 2010 to 2019 affected the Mexican public health system, given the small proportion (0.05%) of hospitalizations and the brief length of hospital stay. The increased number of hospitalizations during the study period may be explained by local and national measures to facilitate foreign residents' access to hospital services, while the decrease in hospital utilization in 2020 is likely associated with COVID-19. Geographic location and the most frequent primary discharge categories of hospitalizations within each population could provide evidence for modifications to public health policy in Mexico.

## Introduction

Mexico is a Latin American country with a long history of being a conduit for irregular migration to the United States (1–4). Recently, its international migration and mobility profile has been changing, slowly evolving into a transit country as well as one which receives persons voluntarily and involuntarily returning from the United States (5, 6). Transit migration through Mexico has traditionally involved individuals from Central America. However, recently, residents from other countries and continents have become more common, and a new mode of migration involving large, organized groups, has emerged (7). These dynamics have contributed to a complex migration profile, which includes temporary and permanent residents, irregular migrants in transit, asylum and refugee seekers, and refugees. The most recent data on the magnitude of mobility show that, in 2019, there were an estimated 1,060,707 foreign nationals with permanent residency in Mexico (0.84% of the national population) (8). Additionally, there were an estimated 182,940 irregular migrants in Mexico, in 2019, although this population experienced a historic drop of 54.42% in 2020 (9). Similarly, the number of asylum seekers dropped 41.68% between 2019 and 2020, from 70,609 to 41,179 (9).

The use of publicly funded health services by foreign residents, especially those with irregular migration status, has been a source of controversy (10, 11). Societal perspectives have ranged from embracing the right to health care for all, to denying publicly funded health services to specific groups, including foreign residents (12). Situated between these two perspectives are policies that support providing certain health services (outpatient care and life-saving urgent care) to specific populations (children and pregnant women), but restrict the provision of high-cost services that can pose financial risk to the health system (12). Stigmatization of foreign residents as disease vectors or as a public health problem can lead to barriers in health care access (10, 13). These views tend to shift based on the sociopolitical climate of the countries of origin, transit, and destination (12).

Within this context, in 2014, the Mexican government laid out a plan to provide temporary (90 days) health insurance coverage for certain services (14). Additionally, in 2019, the government implemented legal changes that recognized the right to health for all people in Mexican territory, regardless of migration status (15). However, the COVID-19 pandemic changed health service offerings and access, leading to a reconfiguration and adjustment of available capacity which had population-level impacts, especially among groups that already faced significant difficulties in accessing hospital services pre-pandemic, such as foreign residents.

Although it is to be expected that Mexico's new migration and mobility profile has led to changes in health care utilization, the magnitude of these changes and the factors that could help explain them is unknown. Scientific evidence is needed to inform decision making and the design of public policy to optimize the use of available resources and, above all, meet the needs of a highly vulnerable population. For this reason, the present study examined factors associated with the distribution and trends of Mexican and foreign resident hospitalizations in Mexican public hospitals from 2010 to 2020 to guide health policies and to enhance foreign residents' health services.

## Materials and methods

This analysis included public hospitals in Mexico, which serve mostly low-income, uninsured populations, including foreign residents. In 2020, these public hospitals covered almost 36% (16) of Mexico's 126 million residents (17). In this descriptive, correlational analysis, we examined 2010–2020 discharge data from Mexican state-run public hospital databases, which is publicly available and generated annually by the Mexican Ministry of Health (18). We analyzed hospital discharge data as a proxy for the evaluation of hospitalizations.

Due to the absence of the code that identifies foreigners, in the variable “place of habitual residence,” it was not possible to include the year 2015 in the analysis. Annual databases consolidate data from official hospital administrative records. They include information on the primary discharge category, coded according to the International Classification of Diseases (ICD-10), and sociodemographic characteristics of users (age, sex, and habitual residence). The databases are open access and are available on the website of the Mexican Ministry of Health (18).

### Data processing and analysis

The final database, composed of 10 annual databases, initially contained 29,971,549 hospitalizations. Once data was cleaned due to discordant and missing values, including the removal of the 2015 data set, 26,780,808 hospitalizations remained. Proportions were estimated for qualitative variables: declared habitual residence [Mexico; the United States (US); Central and South America (CSA); and Other Continents (OC), referring to any country outside Central or South America, the United States, or Mexico], sex, and primary discharge category according to the ICD-10 coding system. To facilitate the presentation of results, Mexican states were grouped into five regions: Central, North-Central, North, Northwest, and South. The median was calculated for numerical and quantitative variables (days in hospital and age) because these variables showed a non-normal distribution; this was proven both graphically and with a Kolmogorov-Smirnov test (*p* < 0.001), 163 which is the recommended test for large samples (19).

We analyzed the distribution of the variables using graphical methods and modeled the relationship between annual hospitalizations and habitual residence, adjusting for sociodemographic characteristics and primary discharge category using Poisson regression (20, 21). We chose this model because the response variable (annual hospitalizations) is a count variable (discrete and non-negative) (22, 23) and can occur at any point in time; additionally, its use to analyze hospital discharges over time has been established in the literature (24). To quantify the impact of omitting 2015, the expected number of hospital discharges of foreign residents was estimated by interpolation. However, each discharge could not be assigned individual characteristics such as place of habitual residence, sex, age, and cause for hospitalization and the estimates could not be included in the analysis.

To perform the analysis using Poisson regression, the database was organized by year, migratory status (foreign or domestic), habitual residence (with four categories described above), sex, age, and primary discharge category, which generated the variable count that identified the number of hospitalizations according to the characteristics of these variables. Crude and adjusted models were analyzed, with year of hospitalization, sex, age, and primary discharge categories as explanatory variables, respectively. Trends were evaluated over the study period by entering the response variable year as continuous in the models. This analysis was repeated, stratifying by sex, age, and primary discharge category for the period 2010–2014 and 2016–2020 for Mexican and foreign residents, respectively. For the analysis of the period 2016–2020, the year 2016 was used as a reference. Temporal trends were calculated by annual number of hospitalizations for Mexican and foreign residents.

#### Ethical considerations

This analysis conforms to all international guidelines on the protection of human subjects. The data used are open access and do not contain personally identifiable information. The analysis protocol was reviewed and approved by the Ethics Committee of the El Colegio de la Frontera Norte (El Colef; 079_230821) as part of a larger project on migration and health in Mexico. This activity was determined by the U.S. Centers for Disease Control and Prevention not to be research as defined in 45 CFR 46.102(l) and IRB review was not required.

## Results

Of the total hospitalizations between 2010 and 2020 (*n* = 26,780,808), 0.05% or 13,277 were of foreign residents (residents of the US, CSA, or OC). As shown in Table 1, 71.42% (*n* = 9,483) of all hospitalizations of foreign residents were among individuals whose habitual residence was OC, and only 3.95% (*n* = 524) were among US residents.

**Table 1:** Sociodemographic and health service characteristics of Mexican and transients and migrants hospitalizations, Mexico, 2010–2020.

	**Mexican** **residents (*n* = 26,767,531)**	**Transients and migrants (*****n*** = **13,277)**			
**Variables**		**Total** **foreign residents**	**U.S.** **(*n* = 524)**	**Central and South** **America (*n* = 3,270)**	**Other Continents (*n* = 9,483)**
Age (median in years)	26.00	30.83	34.50	24.00	34.00
Length of stay (median in days)	2.00	2.00	2.00	2.00	2.00
**Sex (%)**					
Male	27.41	45.46	52.65	37.73	46.00
Female	72.59	54.54	47.35	62.27	54.00
**Primary discharge category (%)**					
Neoplasms and hematologic diseases	6.82	9.70	10.61	7.52	10.29
Circulatory	2.77	4.40	5.87	1.92	5.21
Respiratory	3.96	4.90	7.01	2.16	5.63
Digestive	9.77	9.20	7.20	5.38	10.49
Genitourinary	7.11	7.40	4.36	2.50	9.20
Obstetric	42.45	20.70	5.49	42.24	13.73
Perinatal	4.01	2.60	0.95	3.07	2.67
Trauma injury	7.18	13.10	29.55	22.73	9.27
Others	15.93	28.10	28.98	12.48	33.51
**Geographic region of discharge (%)**					
Central	37.83	9.85	22.51	6.75	0.32
North-Central	16.73	46.98	39.12	3.45	98.37
North	13.72	6.58	18.63	1.01	0.09
Northwest	7.42	3.00	8.62	0.28	0.10
South	24.30	33.59	11.12	88.51	1.12

Source: Hospital discharge database, Mexican Ministry of Health. Office of Health Information (DGIS, for its initials in Spanish), 2010–2020.

The age of Mexican residents hospitalized (Table 1) was similar to the age of CSA residents, with a median age of 26 and 24, respectively; and the median age of US residents approximated that of residents from OC, with a median age of 35 and 34, respectively. The distribution of hospitalizations by sex was higher among females for all categories of habitual residence, except among US residents, where 52.65% of were male. This distribution also varied by category of habitual residence and obstetric hospitalizations were the most common reason for hospitalization among Mexican residents (42.45%), CSA residents (42.24%), and residents from OC (13.73%). In foreign residents, trauma was the second most frequent cause of hospitalization. Also common in both populations were hospitalizations due to causes grouped under “Other,” which included was comprised of 32 individual ICD-10 categories with minor frequencies that summed to ~ 20% of the total number of discharges.

**Figure 1:**
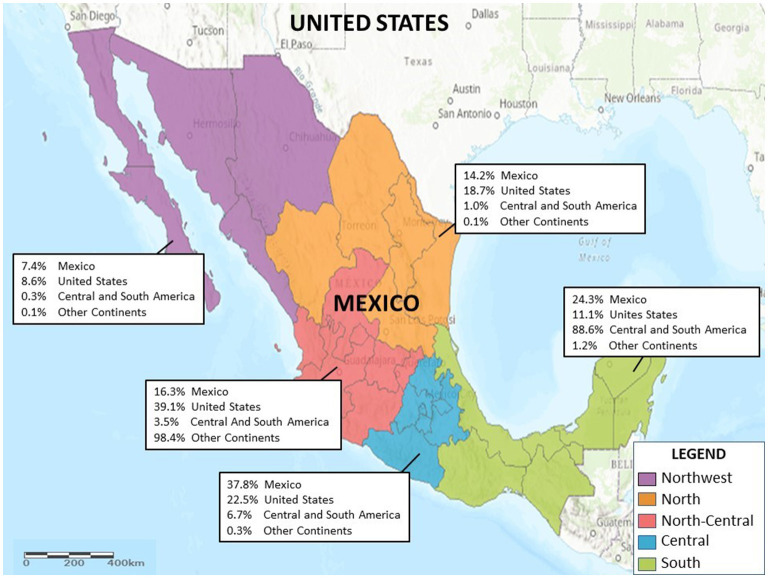
Proportion of hospitalizations by geographic region and habitual residence.

In terms of the geographic region, Table 1 shows that Mexican residents received services mostly (37.83%) in the Central region. CSA residents were hospitalized primarily (88.51%) in the Southern region; US residents and residents from OC were treated mostly (39.12 and 98.37%, respectively) in the North-Central region. There were no observed differences in the length of stay based on habitual residence, and the median length of stay was 2 days. Figure 1 illustrates the hospitalizations by geographic region and habitual residence.

**Figure 2:**
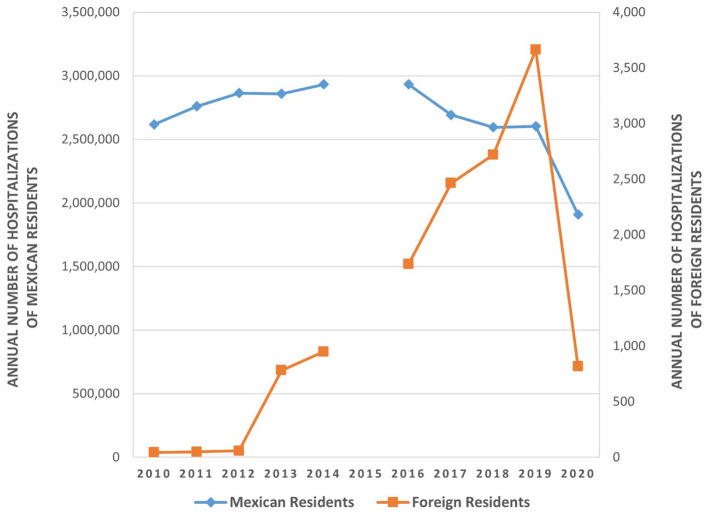
Trends in hospitalizations among Mexican and foreign residents, 2010–2020.

Figure 2 shows the temporal trends of hospitalizations among Mexicans and foreign residents. The annual distribution of hospitalizations of Mexican residents was homogenous, with an annual average of 2.67 million (10.00% per year) and a standard deviation of 0.30 million (1.12% of total hospitalizations). The distribution ranged from a maximum of 2.93 million in 2016 (10.96% of all hospitalizations) to a minimum of 1.91 million in 2020 (7.13% of all hospitalizations). In contrast, the distribution of hospitalizations of foreign residents across the majority of the evaluation period trended upward, with a marked rise in 2019 due to a registered increase of residents from OC. The annual average number of foreign residents hospitalized was 1,300.70 (standard deviation: 1,266.65). However, between 2019 and 2020, there was a pronounced decline among foreign residents (348.8%) as compared with Mexican residents (36.6%).

Table 2 presents the adjusted models using Poisson regression for hospitalizations of Mexican and foreign residents from 2010 to 2014 and 2016 to 2020. Compared to 2010, there was an upward trend in hospitalizations for Mexican residents from 2011 to 2014 and a notable decrease after 2016. On the other hand, hospitalizations of foreign residents trended upward from 2011–2020, compared to 2010, with an increased intensity from 2017–2019. Regarding sex, hospitalizations were higher in females for Mexican residents and higher in males for foreign residents. Both Mexican and foreign residents had a higher frequency of hospitalizations in the ≤ 17 age group, although there was no difference in foreign residents when compared with those over 60 years of age. In both populations, hospitalizations for obstetric causes had the highest frequency, being up to 10 and four times more frequent than traumas in Mexican and foreign residents, respectively. In foreign residents, trauma was the second most frequent cause of hospitalization. Also common in both populations were hospitalizations due to causes grouped under “Other,” which included 32 individual ICD-10 categories with minor frequencies that summed to ~20% of the total number of hospitalizations.

**Table 2:** Adjusted model of Poisson regression analysis of hospitalizations of Mexican and foreign residents, Mexico, 2010–2020.

	**Adjusted models**			
	**Mexican residents**		**Foreign residents**	
	**IR** ^a^	**CI 95%**	**IR**	**CI 95%**
**Year**				
2010	Ref.		Ref.	
2011	1.05	1.05, 1.06	1.14	0.76, 1.71
2012	1.09	1.09, 1.10	1.11	0.75, 1.64
2013	1.09	1.09, 1.09	5.98	4.41, 8.11
2014	1.04	1.04, 1.04	5.72	4.23, 7.74
2016	1.05	1.05, 1.05	8.80	6.52, 11.87
2017	0.79	0.79, 0.79	12.16	9.02, 16.39
2018	0.98	0.98, 0.98	14.13	10.49, 19.04
2019	0.86	0.86, 0.86	19.07	14.17, 25.68
2020	0.56	0.56, 0.56	6.13	4.52, 8.30
Tendency	0.96	0.96, 0.96	1.18	1.17, 1.19
**Sex**				
Female	Ref.		Ref.	
Male	0.69	0.69, 0.69	1.19	1.14, 1.24
**Age (years)**				
< 17	Ref.		Ref.	
18–24	0.74	0.74, 0.74	0.91	0.87, 0.96
25–39	0.88	0.88, 0.88	0.77	0.73, 0.81
40–59	0.59	0.59, 0.59	0.87	0.82, 0.91
60–100	0.64	0.64, 0.65	1.01	0.96, 1.07
**Primary discharge category**				
Trauma	Ref.		Ref.	
Neoplasia	0.95	0.95, 0.96	1.05	0.97, 1.12
Circulatory	0.39	0.38, 0.39	0.58	0.53, 0.64
Respiratory	0.55	0.55, 0.55	0.56	0.51, 0.61
Digestive	1.36	1.36, 1.36	0.93	0.86, 1.00
Genitourinary	0.99	0.99, 0.99	0.89	0.82, 0.96
Obstetric	10.22	10.21, 10.24	4.54	4.26, 4.84
Perinatal	0.71	0.71, 0.72	0.77	0.68, 0.86
Other	2.21	0.71, 0.72	2.07	1.95, 2.19

Adjusted models included all variables presented in the table and their results were obtained considering the variable year as categorical.

a Incident Ratio.

## Discussion

The long history of Mexican migration to the United States has been examined from various perspectives. Previous studies have considered the role of climate change in catalyzing migration, especially from rural areas (1); family and social networks in the United States and the desire for reunification as a motivating factor for migration (25); and worsening sociopolitical and economic conditions, which has long been used to explain international migration is not unique to Mexican society (4). These undercurrents are present in most Latin American countries and could help contextualize the current migration dynamics posing challenges to the Mexican health system (26).

According to the results of this analysis, which incorporates a decade of discharge records in Mexico, the volume and proportion of foreign resident hospitalizations among all hospitalizations was remarkably low (0.05%) over the analysis period. Despite the upward trend in hospitalizations of foreign residents since 2011 and most notably from 2017 to 2019, this increase was unlikely to have had an impact on the health system because the absolute number of hospitalizations remained low, and the median hospital stay was short (2 days). The most significant increase was observed in 2019, possibly due to the implementation of a new national policy (15), which extended health coverage to all persons in Mexican territory, including migrants. The prior policy, in place from 2015 to 2018 only provided 90 days of limited coverage for specific health problems (14), such as treatment for urgent health needs (e.g., childbirth or injuries due to external causes). These findings align with studies carried out in other countries (12, 27).

The COVID-19 pandemic revealed access differences related to social vulnerability (31). Our results showed a general reduction in hospitalizations during 2020. This decrease was slightly higher among foreign residents as compared with the Mexican population. The reduction in foreigner discharges could be attributable to the significant reduction in irregular migration through Mexico to the United States (9) and in the number of individuals seeking refugee status and asylum in Mexico (9). The decreased entry of migrants into Mexico could be related to international travel restrictions, despite the fact that Mexico did not adopt measures restricting mobility (28).

There were differences when comparing hospitalizations among all categories of habitual residence and those limited to foreign residents. Analysis of all hospitalizations showed a higher frequency among younger age groups. These results are similar to the findings by Klein and von dem Knesebeck (27). In relation to sex, a difference in trends was observed according to location of habitual residence; Mexican women and foreign men presented a higher frequency of hospitalizations. These findings are equivalent to those obtained in a 2021 study in Spain which found that the sex of hospital service users varied significantly by region of origin, and that Spanish women used 1.42% more health services than men, while African women's utilization was 18.2% lower (12). Similarly, Waure et al. found that women use health services more frequently than men (29). In contrast, Klein and von dem Knesebeck, found that migrant women use fewer cancer screening services (27). When the results of our analysis were limited to foreign residents, the probability of hospitalizations was higher for men, which aligns with the findings of Gimeno-Feliu in 2021 (12).

The distinct utilization pattern by geographic region could be used to develop a focused health policy in Mexico. CSA residents were overwhelmingly hospitalized in the southern states of Mexico, where the busiest points of entry for migrant populations to the country are located. Six out of ten persons discharged were women, and 43% were treated for obstetrical causes (pregnancy, childbirth, and puerperium). The relatively high frequency of hospitalizations for trauma injuries among CSA could reflect the situation of violence in Mexico (30) and the dangerous conditions of migration (31, 32), which has been documented in other studies (5).

This regional profile of health problems suggests the importance of measures to improve response capacity in the southern states of Mexico. Along the same lines, it is worth noting is that 47.0% of all foreign residents and 98.4% of residents from OC were treated in the hospitals in a single geographic region in Mexico (North Central; Table 1). Understanding this distribution requires other types of analysis that are not possible with the currently available discharge data.

This analysis helps explore the trends, relative burdens, profiles of hospitalizations, geographic distributions, and causes of hospital care for foreign residents in Mexico and for Mexican residents. However, several limitations should be noted. The analysis was based on administrative information generated by the Mexican Ministry of Health, which was the only available source of data on hospitalized foreign residents in Mexico. This constitutes an important limitation to carrying out a more detailed analysis due to the insufficient quantity and type of variables included in the records. In addition, in the case of the variable “place of habitual residence,” the code that identified foreign residents in 2015 was missing. However, the behavior of the surrounding years (2014 and 2016) does not show significant differences in the number or composition of discharges, for Mexicans or foreign residents. Although it would be ideal to have an uninterrupted series of data, the lack of 2015 data did not affect the estimated tendency in the current study. Furthermore, no epidemiological or social event occurred in 2015 that could have modified the sociodemographic, migratory, or health characteristics of foreign residents in Mexico. For this reason, it was assumed that the findings regarding factors associated with hospitalizations can be applied to the omitted year. Foreign residents, especially those from the United States border regions, may have used private care in Mexico, and this data that is not captured by the Mexican public health system. Another element to consider was the low frequency of hospitalizations in the North and Northwest regions of the country (only 1.28% of all hospitalizations), which could suggest substantial underreporting because many foreign residents in Mexico's northern border region seek care in in the United States (33). Lastly, the observed trends are solely based on data from 2010 to 2020. Registry and surveillance systems should be improved to better track the use of health services by foreign residents to inform and evaluate the implementation of national policies aimed at these groups in Mexico.

In reference to the need for policies that facilitate migrants' access to health services, several Latin American countries have implemented national programs to guarantee health services for migrants (34), which could be used to inform efforts in Mexico. There is little evidence in the literature on the effectiveness of these policies. However, Chile provides one of the limited exceptions where a national policy composed of various strategies at the regulatory and financial level has been implemented to eliminate legal and economic barriers to health services for migrants (35). One strategy involved the health system hiring migrants who spoke languages other than Spanish to reduce linguistic and sociocultural barriers (36). Another strategy involved social communication campaigns aimed at reducing or eliminating xenophobia and stigmatization through education about the contribution of migrants to the economic and social wellbeing of the country [4.00% of the 2019 Gross Domestic Product was attributed to this population group (37)]. The effects of these strategies in the elimination of access barriers were seen in the gradual increase in the migrants' use of outpatient and hospital services covered by the National Health Insurance (FONASA). This contributed to a significant reduction (72.00%) in the proportion of hospital discharges without FONASA coverage, which resulted in a decrease in impoverishing out of-pocket expenses for migrants (38).

In this context, mechanisms to facilitate migrants' access to health services in Mexico should be implemented. These efforts would support Mexico's 2019 policy authorizing health services for all persons regardless of migratory status.

## Data availability statement

Publicly available datasets were analyzed in this study. This data can be found at: http://www.dgis.salud.gob.mx/contenidos/basesdedatos/da_egresoshosp_gobmx.html.

## Author contributions

RL-F contributed to the design, writing, and final revision of the document. BA-G and MP-S contributed to the design, cleaning, organization, construction of the base, performed the analysis, writing, and final revision of the document. IB, NB, RC-A, and DG-L contributed to the development and editing of the document. All authors contributed to the article and approved the submitted version.
